# Deterioration in Mental Health Under Repeated COVID-19 Outbreaks Greatest in the Less Educated: A Cohort Study of Japanese Employees

**DOI:** 10.2188/jea.JE20200499

**Published:** 2021-01-05

**Authors:** Natsu Sasaki, Reiko Kuroda, Kanami Tsuno, Kotaro Imamura, Norito Kawakami

**Affiliations:** 1 Department of Mental Health, Graduate School of Medicine, The University of Tokyo, Tokyo, Japan; 2 Division for Environment, Health, and Safety, The University of Tokyo, Tokyo, Japan; 3 School of Health Innovation, Kanagawa University of Human Services, Kanagawa, Japan

Kikuchi et al reported that an increase in psychological distress among low-income people in Japan from February to April 2020^1^ coincided with the 1^st^ wave of the COVID-19 pandemic (16 January–31 May 2020).^2^ In Japan, the number of suicides in August 2020 was much higher than in the same month the previous year,^3^ although the numbers from February to June 2020 were down more than 10 percent from the previous year. We speculated that the 2^nd^ wave of the COVID-19 pandemic (1 June–19 August 2020) may show the accumulation of mental health deterioration in the community. In confirmation, we showed a change in psychological distress from the end of the 1^st^ wave to the end of the 2^nd^ using a cohort of full-time employees in Japan. Further, we replicated the study to investigate psychological distress among people with low socioeconomic status (SES).

**Figure 1.**
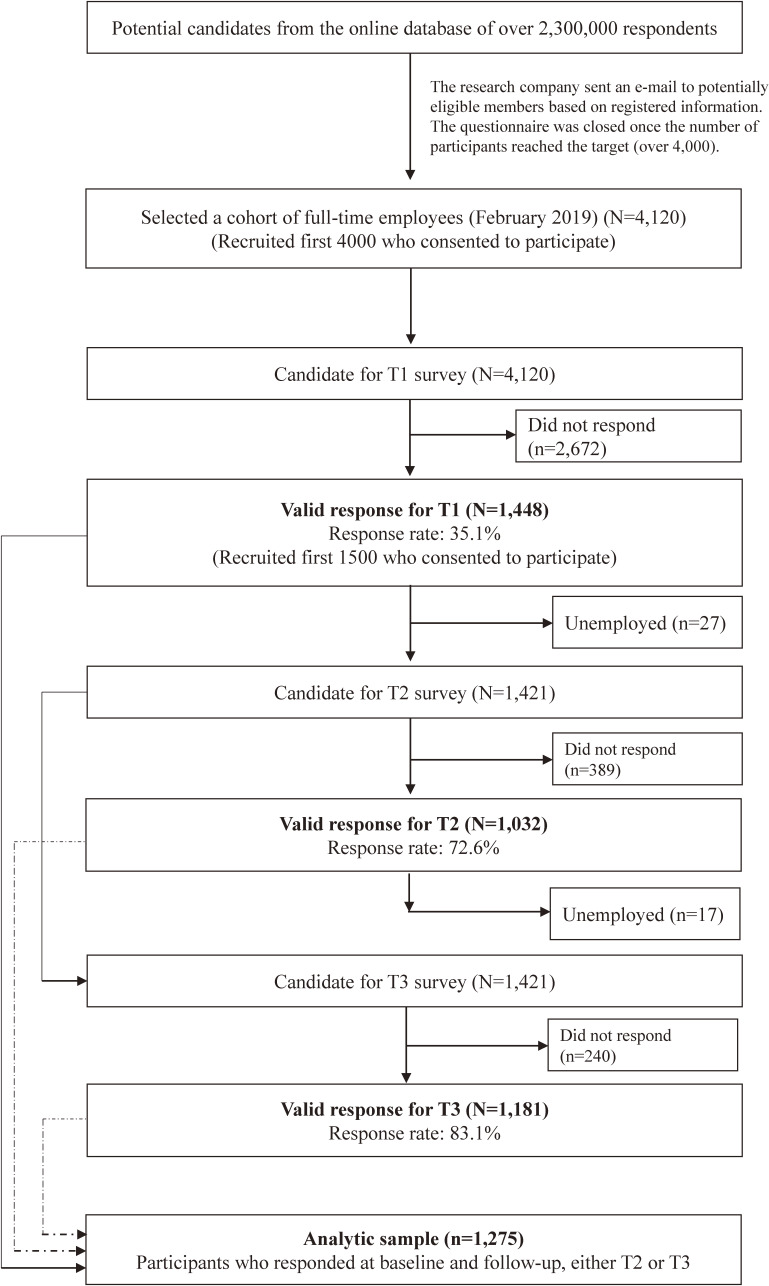
Flowchart of participant recruitment.

We conducted a longitudinal survey extracting a sample from the cohort of full-time employees in Japan (*n* = 4,120; created in February 2019). Participants were recruited from enrollees of a research company, which sent an e-mail to potentially eligible members based on registered information. The questionnaire was closed once the number of participants reached the target (over 4,000). Participants were invited to complete an online survey on March 19–22, 2020 (T1). A total of 1,448 participated at T1. Respondents, excluding unemployed (*n* = 21), were again invited to complete the survey on 22–26 May 2020 (T2) and on 7–12 August 2020 (T3).^4^ Figure 1 depicts the flowchart. Participants were assured of their anonymity and asked to sign an informed consent online. This study was approved by the Research Ethics Committee of The University of Tokyo (No. 10856-(2)(3)(4)). Psychological distress in the last 30 days was measured with an 18-item subscales of the Brief Job Stress Questionnaire (BJSQ).^5^ Educational attainment, measured at T2 and T3, was dichotomized into ≥16 years (high) or less (low). A mixed-model repeated-measures analysis of variance with an unstructured covariance matrix was conducted using group (high or low education) × time (T1, T2, T3) interaction as an indicator of group differences. Analyses were adjusted for age category, gender, and marital status. Participants who responded to the baseline and follow-up survey, either at T2 or T3, were included in the analysis (*N* = 1,275, low [*n* = 607] and high education [*n* = 668]) (Table 1). The outcome variable (ie psychological distress) was treated as missing if participants were laid off at T2 or T3 in the mixed-model analysis. The results showed that low education was associated with a significant increase in psychological distress from T1 to T3 compared to high education (adjusted estimates of fixed effect 1.26 [95% CI 0.28–2.24], *P* = 0.012). Details are shown in Tables 2 to 4.

**Table 1.** Baseline characteristics of participants stratified by level of educational attainment (*N* = 1,275)

	Low education^a^ (*n* = 607)		High education^a^ (*n* = 668)		*P* for difference^b^

	*N* (%)	Mean (SD)	*N* (%)	Mean (SD)	
Gender					<0.001
Male	245 (40.4)		401 (60.0)		
Female	362 (59.6)		267 (40.0)		
Age		43.2 (10.6)		40.0 (10.2)	<0.001
20–29 years old	92 (15.2)		139 (20.8)		
30–39 years old	136 (22.4)		210 (31.4)		
40–49 years old	161 (26.5)		168 (25.1)		
50–59 years old	218 (35.9)		151 (22.6)		
Marital status					
Single	322 (53.0)		304 (45.5)		0.007
Married	285 (47.0)		364 (54.5)		
Occupational type					<0.001
Managers	26 (4.3)		89 (13.3)		
Nonmanual	345 (56.8)		458 (68.6)		
Manual	236 (38.9)		121 (18.1)		
Company size					<0.001
>1,000 employees	144 (24.5)		267 (41.1)		
300–999	91 (15.5)		118 (18.2)		
50–299	167 (28.4)		173 (26.6)		
<50	186 (31.6)		92 (14.2)		

SD: standard deviation.

^a^Educational attainment, measured on T2 or T3, was dichotomized into ≥16 years (high) or less (low).

^b^*P* value for difference was calculated by the chi square test for variables, except for age, for which the *t* test was used.

**Table 2.** Crude means of psychological distress^a^ at baseline (T1), T2 and T3 under the COVID-19 pandemic among a cohort of Japanese full-time employees (*N* = 1,275)

Survey (time of survey)	Total *N*	Low Education^b^			High Education^b^		

		*n*	Mean	SD	*n*	Mean	SD
T1 (March 2020)	1,275	607	41.0	11.2	668	41.2	11.5
T2 (May 2020)	968	454	41.5	11.3	514	41.0	10.9
T3 (August 2020)	1,148	541	42.7	11.7	607	41.4	11.5

COVID-19: Coronavirus disease 2019.

SD: standard deviation.

^a^Psychological distress was measured using a scale from the Brief Job Stress Questionnaire (BJSQ). Scores ranged from 18 to 72, with a higher score indicative of higher distress. Cronbach’s alpha coefficients of the scale ranged from 0.93–0.94 among the T1, T2, and T3 surveys.

^b^High education was defined as ≥16 years of educational attainment (ie, university graduate or higher).

**Table 3.** Crude and adjusted estimated mean of psychological distress^a^ at baseline (T1), T2 and T3 under the COVID-19 pandemic among a cohort of Japanese full-time employees: mixed model with repeated measures (*N* = 1,275)

Survey (time of survey)	Estimated means (SE)			
	Crude		Adjusted^b^	
	
	Low Education^c^	High Education^c^	Low Education^c^	High Education^c^
T1 (March 2020)	41.0 (0.5)	41.2 (0.4)	41.2 (0.5)	41.3 (0.4)
T2 (May 2020)	41.5 (0.5)	40.8 (0.5)	41.7 (0.5)	40.9 (0.5)
T3 (August 2020)	42.5 (0.5)	41.4 (0.5)	42.6 (0.5)	41.5 (0.5)

SE: standard error.

^a^Psychological distress was measured using a scale from the Brief Job Stress Questionnaire (BJSQ). Scores ranged from 18 to 72, with a higher score indicative of higher distress. Cronbach’s alpha coefficients of the scale ranged from 0.93–0.94 among the T1, T2, and T3 surveys.

^b^Adjusted for age, gender, and marital status.

^c^High education was defined as ≥16 years of educational attainment (ie, university graduate or higher).

**Table 4.** Excess effects of low educational attainment on psychological distress^a^ across three consecutive surveys (T1-T3) of full-time employees in Japan during the COVID-19 pandemic: mixed model with repeated measures (*N* = 1,275)

	Comparison between surveys	Estimates of fixed effects for time × education^b^ interaction	SE	95% CI	*t*	*P*
Crude	T1-T2	0.95	0.53	−0.095–2.00	1.79	0.075
	T1-T3	1.26	0.50	0.28–2.24	2.53	0.011
Adjusted^c^	T1-T2	0.93	0.53	−0.12–1.98	1.74	0.082
	T1-T3	1.26	0.50	0.28–2.24	2.53	0.012

CI: confidence interval SE: standard error.

T1-T3: surveys at baseline (March 2020) and at follow-up (May and August 2020), respectively.

^a^Psychological distress was measured using a scale from the Brief Job Stress Questionnaire (BJSQ). Scores ranged from 18 to 72, with a higher score indicative of higher distress. Cronbach’s alpha coefficients of the scale ranged from 0.93–0.94 among the T1, T2, and T3 surveys.

^b^Educational attainment was scored as 0 for high (≥16 years, ie, university graduate or higher) and 1 for low.

^c^Adjusted for age, gender, and marital status.

Our survey has several limitations. It omitted some important socioeconomic variables (eg, household income, housing tenure), and its generalizability is limited because it included only full-time employees. However, the results suggested that psychological distress among employees with low education levels has worsened through the end of the 2^nd^ wave of the pandemic, supporting a previous report.^1^ People with low SES, especially those with low education, maybe be at increased risk of infection and psychological stress. They have several factors that may increase their exposure to COVID-19, such as limited job opportunities, which do not allow them to work from home (eg, wholesale, transportation),^6^ and limited access to reliable information about prevention. In this cohort, low education workers likely belong to smaller companies, which have been shown to implement less preventive measures for COVID-19.^7^ Besides the infection, they may experience a psychological burden, such as the negative economic effects of COVID-19 control measures (ie, temporarily forced to leave employment or to reduce working hours) on the household due to their unstable work conditions, harassment related to COVID-19, and fewer social resources. In Japan, low education attainment predicts the risk of suicide.^8^ To promptly reduce their psychological burden during COVID-19, it is important to close the knowledge gap in COVID-19 situations: providing trustworthy and easily understandable information about how to prevent infections, how to get financial support, and how to maintain mental health.
